# Three-year trajectories of global perceived quality of life for youth with chronic health conditions

**DOI:** 10.1007/s11136-016-1353-z

**Published:** 2016-07-05

**Authors:** Janette McDougall, David J. DeWit, Megan Nichols, Linda Miller, F. Virginia Wright

**Affiliations:** 1 Thames Valley Children’s Centre, 779 Baseline Road East, London, ON N6C5Y6 Canada; 2 Western University, 1151 Richmond Street, London, ON N6A 3K7 Canada; 3 Bloorview Research Institute, 150 Kilgour Road, Toronto, ON M4G 1R8 Canada

**Keywords:** Quality of life, Self-report, Youth, Chronic conditions, Latent class growth analysis, Change trajectories

## Abstract

**Purpose:**

Objectives of this longitudinal study were to examine 3-year trajectories of global perceived quality of life (QOL) for youth with chronic health conditions, as obtained from youth and parent reports, and to identify personal and environmental factors associated with the trajectory groups for each perspective.

**Methods:**

Youth with various chronic conditions aged 11–17 years and one of their parents were recruited from eight children’s treatment centers. Latent class growth analysis was used to investigate perceived QOL trajectories (separately for youth and parent perspectives) over a 3-year period (four data collection time points spaced 12 months apart). Multinomial logistic regression was employed to identify factors associated with these trajectories.

**Results:**

A total of 439 youth and one of their parents participated at baseline, and 302 (69 %) of those youth/parent dyads completed all four data collection time points. Two QOL trajectories were identified for the youth analysis: ‘high and stable’ (85.7 %) and ‘moderate/low and stable’ (14.3 %), while three trajectories were found for the parent analysis: ‘high and stable’ (35.7 %), ‘moderate and stable’ (46.6 %), and ‘moderate/low and stable’ (17.7 %). Relative to the ‘high and stable’ groups, youth with more reported pain/other physical symptoms, emotional symptoms, and home/community barriers were more likely to be in the ‘moderate and stable’ or ‘moderate/low and stable’ groups. Also, youth with higher reported self-determination, spirituality, family social support, family functioning, school productivity/engagement, and school belongingness/safety were less likely to be in the ‘moderate and stable’ or ‘moderate/low and stable’ groups, compared to the ‘high and stable’ groups.

**Conclusion:**

Findings suggest that youth with chronic conditions experience stable global perceived QOL across time, but that some individuals maintain stability at moderate to moderate/low levels which is related to ongoing personal and environmental influences. Potential benefits of universal strategies and programs to safeguard resilience for all youth and targeted interventions to optimize certain youths’ global perceived QOL are indicated.

**Electronic supplementary material:**

The online version of this article (doi:10.1007/s11136-016-1353-z) contains supplementary material, which is available to authorized users.

## Introduction

Over the last few decades, quality of life (QOL) research for children and youth with chronic health conditions (i.e., long-term diseases, disorders, injuries, and related health problems [1, 2]) has been increasing and evolving. The standard approach in this area has been to study QOL as a health-related, multidimensional concept, mainly involving family and service provider reports of children’s physical, psychological, and social functional status [3]. This concept of health-related QOL (HRQOL) emerged partially due to the broader definition of health proposed by the World Health Organization 70 years ago, which describes health as a ‘state of complete physical, mental, and social well-being and not merely the absence of disease or infirmity’ (p. 100) [4]. Although this development was progressive and expanded the aspects of health/function being evaluated by the healthcare field, another result has been that QOL is often conceptualized and measured in terms of health-related concepts [5].

A contemporary review [6] of patient-reported outcome measures concluded many QOL instruments were developed before clarification of conceptual differences between functioning, disability, health, and QOL was provided by the World Health Organization’s (WHO) International Classification of Functioning, Disability and Health (ICF) [1], and the WHO-QOL Group [7]. This group defined QOL as, ‘individuals’ *perceptions* of their position in life in the context of culture and value systems in which they live, and in relation to their goals, expectations, standards, and concerns’ (p. 1570) [7]. Interest has grown in measuring QOL as subjective well-being from the perspectives of children and youth [8, 9]. Subjective well-being has been thought to have multiple constructs, i.e., positive affect, negative affect, domain satisfactions, global life satisfaction [10]. WHO’s Regional Office of Europe [11] recommends that countries include a global life satisfaction measure in national surveys, indicating WHO’s support for this concept as relevant to people’s well-being and for societal improvement.

A number of qualitative studies in the past 15 years have gathered perspectives of children and youth with chronic conditions about what is important to their QOL (see for a review [12]). Only one [12], however, asked youth what QOL meant to them. They overwhelmingly defined QOL as an overall sense of life satisfaction or enjoyment. Other qualitative studies of childhood cancer survivors and youth in the general population showed similar findings [13, 14]. This work suggests QOL is viewed by children and youth as a global rather than a multidimensional concept and lends credence to researchers [15] who contend that ‘It is entirely consistent to claim QOL is both unidimensional and multiply caused’ (p. 23).

In consideration of all this, one approach that assesses QOL subjectively and avoids confounding the measurement of QOL with the measurement of its potential correlates in population-based research is to measure it as a unidimensional construct, in terms of a person’s overall life satisfaction or global perceived QOL, and then examine its association with those correlates [16]. Much cross-sectional research has been conducted examining correlates of global perceived QOL for children and youth in the general population [16]. Indeed, these survey-based studies have found significant personal and environmental factors related to this outcome for typically developing young people [17–20]. However, few studies have taken this approach with children and youth who have chronic conditions.

Chong et al. [21] used the Student Life Satisfaction Scale (SLSS) [22], a measure of global perceived QOL with a sample of 48 children aged 8–18 years to examine how children’s perceptions of their cerebral palsy affected their life quality. The SLSS consists of seven items such as ‘My life is going well’ and ‘I wish I had a different kind of life.’ Results showed lower levels of concern about, and fewer perceived consequences of their cerebral palsy as significantly related to higher QOL. Emerson et al. [23] used a representative sample of Australians aged 15–29 years from a national survey to explore cross-sectional associations between having a chronic condition, disability, or impairment, social/emotional support, hardship, and global perceived QOL. QOL was measured using a single survey item, ‘All things considered, how satisfied are you with your life?’ A factorial analysis of variance suggested lower QOL is not inherently related to disability status, but is associated with social exclusion and material hardship. A cross-sectional analysis of baseline data from the present longitudinal study [24] explored multiple personal and environmental factors related to global perceived QOL for 439 youth aged 11–17 years with a chronic condition. QOL was rated by each youth and one parent on an abbreviated SLSS. Linear regression was used with a set of validated measures to identify significant correlates of QOL. Positive correlates were spirituality, school productivity and engagement, family social support, family functioning, and school belongingness/safety, while negative correlates were pain/other physical symptoms, emotional symptoms, including social anxiety, and environmental barriers (home, school, community).

Longitudinal studies that track global perceived QOL of children and youth with chronic conditions are rare. One study examined changes in this outcome for 67 children and youth aged 7–18 years with a life-threatening illness over a 6-month follow-up [25]. The SLSS was again used to measure QOL. Health-related functioning problems (e.g., pain) were related to negative changes in QOL, while benefit finding (e.g., potential benefits of illness) and character strengths (e.g., vitality) were associated with positive changes. Researchers in Belgium examined factors linked with changes over time in global perceived QOL for 429 adolescents with congenital heart disease aged 14–18 years, using a linear analogue scale from 0 (‘worst life’) to 100 (‘best life’) [26, 27]. Over an 18-month follow-up, findings showed both depressive symptoms and loneliness were negatively related, and paternal support positively related, to QOL changes [27]. Emerson et al. [28] used the same Australian survey and sample as described above, this time employing concurrent and historical data for three previous waves and using propensity score matching to determine whether prior exposure to adversity and access to resources were related to self-reported disability and subjective well-being. Previous disability was associated with lower subjective well-being. However, when between-group differences in social context were controlled for, differences in subjective well-being were eliminated.

### Study purpose

Given the clear need to understand patterns in global perceived QOL over time for youth with chronic conditions, this research used the longitudinal data gathered from both youth and one of their parents from the above-mentioned study by McDougall et al. [24] to assess any change in this outcome for youth and to identify key factors associated with that change over a 3-year follow-up. Assessments of child well-being should first take into account children’s own perspectives [29]. However, perspectives of parents are also helpful for making both intervention- and policy-related decisions since parents are likely to have unique insights into their children’s lives and to place different values on life states [30, 31].

Due to the considerable variation in baseline QOL scores [24], the possibility of the existence of distinct group trajectories of change was important to consider. Distinctions in group trajectories cannot be revealed by modeling a single average trajectory. Identifying various trajectories of global perceived QOL that these youth may experience extends on previous studies by ascertaining groups at highest risk, and the unique factors by which they become vulnerable. While this approach has been used to examine HRQOL trajectories for children with specific conditions such as new-onset epilepsy [32], to the authors’ knowledge, it has not been used to examine trajectories of global perceived QOL for a sample of youth with various chronic health conditions. Much research supports a ‘non-categorical’ approach where children with different chronic conditions are combined into a group for data analysis purposes given the commonality in their psychological, psychosocial, and social implications [33, 34].

Specific objectives were to: (1) identify distinct trajectories of both youth and parent reports of youths’ global perceived QOL over 4 time points spaced 12 months apart and (2) examine factors associated with group trajectories of both youth and parent reports of youths’ QOL over that same time period. The overall approach to studying QOL is based on a systems perspective as depicted in a modified version of the WHO ICF Model of Functioning and Disability. The original ICF model [1] shows an individual’s functioning as an interaction among his/her health condition and contextual (personal and environmental) factors. The modified model expands on the ICF model and depicts a person’s perceived QOL and his/her potential for development as the outcomes and ongoing processes arising from the interconnected, ever-changing influences of health, functioning, and contextual factors (see [35]).

## Methods

Youth were recruited from eight children’s treatment centers across Ontario, Canada. A prospective cohort design [36] was used, with a 3-year follow-up. Youth were randomly selected with replacement (i.e., if a randomly selected person declined participation, another person was randomly selected to take his/her place) from lists of potential participants compiled at each center using a computerized randomization method. One parent participated for each youth enrolled. The parents themselves decided which one of them would participate. Youth between the age range of 11 and 17 were included in the study. They had one of the following as their primary condition: cerebral palsy, spina bifida, autism spectrum disorder, acquired brain injury, developmental delay, cleft lip and/or palate, Down syndrome, arthritis, communication disorder, amputation, or any other non-progressive muscular or central nervous system disorder. To be eligible, the youth also needed to be able to cognitively understand and answer the study questionnaire with guidance from a study interviewer. Youth with any progressive conditions (e.g., muscular dystrophy) were excluded since a significantly decreased life span and impact of rapid deterioration may have a different effect on global perceived QOL over time.

Baseline and subsequent data collection occurred either in a private office at the youth’s treatment center or in the youth’s home. Study interviewers were health professionals (e.g., occupational therapists, physical therapists, speech-language pathologists) based at each center trained in the interview protocol. The interviewers obtained written informed assent/consent from youth/parents, respectively, before baseline assessment. Each youth participated in a face-to-face guided questionnaire completion process (30–60 min). A parent questionnaire (30–60 min) was completed independently in a separate room at the same time as the youth interview. The same questionnaires were completed at 12, 24, and 36 months. Families who wished to drop out of the study were not called for further follow-ups. Youth and/or parents who were not able to complete a follow-up at a particular time point (for example, due to prolonged illness, repeated interview cancellations) were still contacted the following year to set up their next follow-up. Overall study ethical approval was obtained from the Health Sciences Research Ethics Board, Western University, London, Canada.

### Measures

An abbreviated youth version of the SLSS and an abbreviated parent-worded version of the SLSS were used to measure global perceived QOL across the four time points. These versions showed psychometric strength in the factor analyses with the present data set (see [37]) and include five positively worded items such as ‘My life is just right’/My child’s life is just right,’ measured using a 6-point Likert scale ranging from 6 = strongly agree to 1 = strongly disagree. Cronbach’s alpha for both the youth and parent abbreviated versions of the SLSS was good (*α* = 0.82 and 0.88, respectively), and a one-factor structure was indicated, accounting for 61 % of the variance in the youth version and 69 % in the parent version. The youth and parent factors were moderately correlated (*r* = 0.42, *p* < 0.001). However, youth mean scores were significantly higher than parent mean scores on the abbreviated measure (25.43 vs 23.29, respectively; *t* = 9.06, *p* < 0.001). There were no significant differences in QOL mean scores found for condition groups (*F* = 1.35, *p* = 0.22). For description of the testing of the original and abbreviated measures in the study data set, see McDougall et al. [37].

 To measure personal- and environmental-level factors hypothesized to be associated with QOL and related changes, a set of validated instruments that measured these constructs were selected to comprise the study youth and parent questionnaires. Descriptions of all measures [37–44] and their subscales (constructs) used in this paper, whether the measure is a youth or parent report, number of items per subscale, examples of item content, and Cronbach’s alpha for each subscale at baseline are shown in Table 1. Age at diagnosis and basic socio-demographics (youth and parent age, youth and parent gender, marital status, education, and income) were gathered in the baseline parent questionnaire and included as fixed covariates (control variables).

**Table 1:** Measures and their subscales/constructs used as correlates or outcomes

Measures	Subscales/constructs used in analyses and examples of item content	Report	# Items	*α*
*Correlates*—*youth functioning/personal factors*				
Strengths and Difficulties Questionnaire [38]	Emotional symptoms (e.g., worries a lot; often unhappy)	Youth	5	0.71
Child and Adolescent Factors Inventory [39]	Pain/other physical symptoms (e.g., physical symptoms such as headaches, dizziness, discomfort)	Parent	2	0.60
Spirituality Index [40] (adapted for youth)	Spirituality—defined as deep feelings/beliefs (e.g., spirituality helps to understand life purpose even when there are problems, feels spiritual peace inside)	Youth	4	0.85
School Productivity/Engagement Measure^a^	Personal effort/success at school (e.g., how often completed homework in last month; how often tried to do personal best at school in last month)	Parent	4	0.86
Self-Determination Scale (adapted/abbreviated from *Arc’s* Self-Determination Scale) [41]	Goal orientation (e.g., if wants to do something finds a way to do it; makes plans for the future)	Youth	6	0.69
*Correlates*—*environmental factors*				
Social Support Appraisal Scale [42]	Family support (e.g., family listens to ideas; thinks family cares)	Youth	6	0.83
Family Functioning Scale [43]	General family functioning (e.g., able to make decisions to solve problems; members accepted for who they are)	Parent	6	0.78
Scale of School Environment [44]	School belongingness/safety (e.g., feels like belongs at school; school is safe)	Youth	3	0.77
Child and Adolescent Scale of the Environment [39]	Home and community barriers (e.g., family stress, community attitudes toward child; lack of support, services, and funding)	Parent	9	0.85
*Outcomes*				
Students’ Life Satisfaction Scale [22] Youth—Revised [37]	Overall perceived quality of life of youth from youths’ perspective (e.g., my life is going well; my life is just right)	Youth	5	0.82
Students’ Life Satisfaction Scale Parent—Revised [37]	Overall perceived quality of life of youth from parents’ perspective (e.g., my child feels his/her life is going well; my child feels his/her life is just right)	Parent	5	0.88

^a^Measure developed for study

### Statistical analyses

Latent class growth analysis (LCGA) using M*plus* 6.11 software [45] was performed to identify classes (groups) of youth with unique trajectories of global perceived QOL as measured by the abbreviated SLSS. While traditional growth curve modeling assumes the existence of a single developmental trajectory, LCGA tests if the population can be best represented by two or more groups of individuals sharing unique trajectories. Individual variation around the class-specific mean trajectories is attributed to random error. As a result, the variances of the intercept and slope parameters for each class are constrained to zero, making it a special subcategory of general growth mixture modeling (GMM) [46]. LCGA is often the preferred method for estimating class-specific trajectories when the interest of the investigator lies solely in examining predictors of membership in qualitatively unique trajectories [47]. The models are less complex (i.e., requiring fewer estimated parameters), minimizing the chance of convergence problems and improper solutions [46]. Moreover, in longitudinal cohort studies, LCGA has been found to outperform GMM and other classification techniques in detecting linear trajectories [48].

Since there is not a single best method for identifying the optimal number of classes in growth mixture models, three widely accepted fit statistics were chosen: (1) Bayesian information criterion (BIC); (2) sample size-adjusted BIC (SSA-BIC); and (3) Lo–Mendell–Rubin likelihood ratio test (LMR-LRT) [46]. The BIC and SSA-BIC examine the impact of adding an additional class on the value of the log likelihood while adjusting for the increase in the number of model parameters. Smaller BIC values are generally associated with the better fitting model with differences of 10 or more used to favor one model over another [49]. The LMR-LRT is a measure of relative fit comparing the fit of a model having *k* classes with the fit of a model having *k* − 1 fewer classes. A statistically significant *p* value suggests that the current (*k* class) model is a significant improvement over the model with *k* − 1 classes.

Selection of the optimal model was also guided by two indices of the quality of classification accuracy: (1) entropy, a standardized measure (bounded between zero and one) of an individual’s overall probability of being in the most likely class, and (2) average posterior probabilities for evaluating the accuracy of classification of each class separately. Although there is not a consensus on satisfactory entropy values, values of 0.80 or greater are generally considered to indicate good or acceptable classification quality [50]. However, minimum acceptable thresholds of 0.70 are also common [51]. Typically, average posterior probability values should be >0.70. Other recommended criteria that were used for determining optimal class solutions included model parsimony (favouring less complex models) and a visual inspection of the plausibility of the estimated trajectories [46].

In the conditional LCGAs, factors associated with class membership in the model with the optimal class solution follow a multinomial logistic regression framework with the influence of a given covariate, indicating the likelihood of belonging to one or more classes relative to a reference class, typically the largest normative group. In this study, factors included in the youth and parent models are among those initially hypothesized to be related to youths’ global perceived QOL (see [52]) and found to be significant correlates of youths’ and parents’ reports of this outcome in the baseline analyses (see [24]).

Standard errors in the unconditional and conditional models were adjusted for possible design effects (i.e., non-independence of observations arising from nesting of youth within the eight treatment centers). To make these adjustments, the youth treatment center ID variable was used to define the nesting or cluster variable in M*plus* followed by specification of the COMPLEX subcommand in the analysis statement. Full information maximum likelihood (FIML) was used to handle missing data on dependent variables (youth and parent perceived global QOL). Assuming data are missing at random, FIML yields less biased and more efficient parameter estimates compared with traditional list or pairwise deletion methods [53]. To handle missing data on all baseline covariates, 10 data sets were generated for both the youth and parent analyses using the multiple imputation procedure in IBM SPSS Statistics, version 23. Rather than read these stacked data sets in M*plus* to produce one set of pooled LCGA results, one imputed data set was randomly selected from SPSS for each analysis and LCGA was performed in M*plus* on the single data set (see [54, 55]). This approach was taken because the analysis of multiple imputed data sets in M*plus* often results in switching of the ordering of classes from one data set to the next, making it impossible to carry out LCGA based on results that are pooled across data sets [56]. To handle multivariate non-normal data, the maximum likelihood estimator (MLR) for generating robust standard errors was chosen.

## Results

### Initial response rates and follow-up attrition

Four hundred and thirty-nine youth and one of their parents completed the baseline questionnaire. The overall initial list of potential participants across centers consisted of 3188 youth. Three hundred and ninety-three of these youth could not be contacted. Of those contacted, 1372 were deemed ineligible (see criteria above) and 984 declined to participate (no interest, busy, youth acutely ill, other), leaving 439 youth–parent dyads who agreed to participate. Participating families did not differ significantly on a number of socio-demographic characteristics (i.e., gender, language spoken in the home, education, rural/urban place of residence) from those who declined to participate. An exception was parent age with participating families having a mean parent age (*M* = 44.84, SD = 6.53) higher than that for non-participating families (*M* = 42.73, SD = 10.35) [*F*(1, 693) = 10.85, *p* = 0.001].

Follow-up questionnaire completion rates were as follows: 88 % (385: 376 parent/youth, 4 youth-only, and 5 parent-only) at 12 months; 83 % (363: 350 parent/youth, 8 youth-only, and 5 parent-only) at 24 months; and 80 % (351: 328 parent/youth, 16 youth-only, and 7 parent-only) at 36 months. In 69 % of families (302/439), both youth and parent completed all 4 time points. To identify systematic sources of attrition, logistic regression analyses were performed in which baseline socio-demographic and health-related variables were entered as multivariate correlates of youth and parent non-completion of at least one follow-up interview and of youth and parent non-completion of all follow-up interviews. No significant factors related to either youth or parent non-completion emerged, suggesting data were missing at random.

### Sample characteristics at baseline

Slightly more than half of the baseline sample of youth were male (55.4 %). The mean age of youth at baseline was 13.7 (SD = 2.2). Cerebral palsy (34.3 %) was the most prevalent condition. See Table 2 for additional sample characteristics.

**Table 2:** Description of study sample characteristics at baseline

Characteristics	*n*	Percent	*M*	SD	Min–max
Youth gender					
Female	193	44.0	–	–	–
Male	246	56.0	–	–	–
Youth age (years)	439	–	13.7	2.2	11–17
Youth primary chronic health condition					
Cerebral palsy	153	34.9	–	–	–
Spina bifida	36	8.2	–	–	–
Autism spectrum disorder	38	8.6	–	–	–
Brain injury	59	13.4	–	–	–
Cleft lip–palate/communication	41	9.4	–	–	–
Developmental delay	29	6.6	–	–	–
Other condition (i.e., amputee, arthritis, Down syndrome, other central nervous system or neuromuscular disorder)	83	18.9	–	–	–
Parent gender					
Female	386	87.9	–	–	–
Male	53	12.1	–	–	–
Parent age (years)	439	–	44.8	6.5	29–71
Parent marital status					
Married	294	67.0	–	–	–
Living common law/partner	38	8.7	–	–	–
Separated/divorced/widowed	76	17.3	–	–	–
Single (never married)	27	6.2	–	–	–
Missing data	4	0.8	–	–	–
Parent education					
Secondary school or less	28	6.4	–	–	–
Completed secondary school	65	14.8	–	–	–
Some college or university	85	19.4	–	–	–
Completed college or university	257	58.5	–	–	–
Missing data	4	0.8	–	–	–
Family income					
Under $25,000	62	14.1	–	–	–
$25,000–$34,999	32	7.3	–	–	–
$35,000–$44,999	32	7.3	–	–	–
$45,000–$54,999	29	6.6	–	–	–
$55,000–$64,999	28	6.4	–	–	–
$65,000–$74,999	42	9.6	–	–	–
$75,000 or more	161	36.7	–	–	–
Missing data	53	12.0	–	–	–

### Unconditional models

**Fig. 1:**
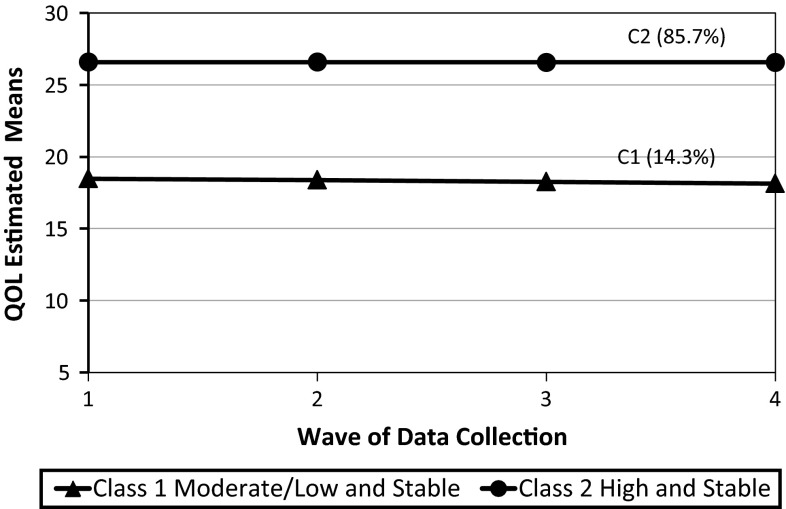
Unconditional model of distinct trajectories for two-class model—youth report

**Fig. 2:**
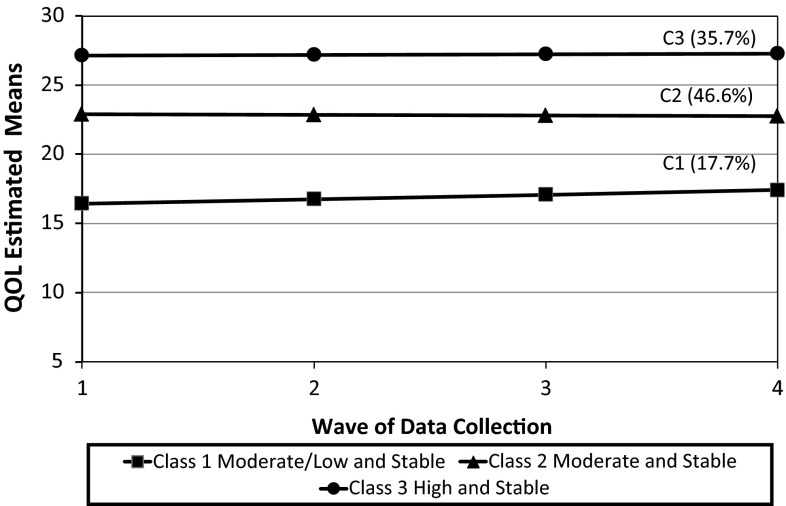
Unconditional model of distinct trajectories for three-class model—parent report

As a preliminary step in the estimation of the unconditional models, both linear and quadratic trajectories were examined. Results supported a linear specification for youth and parent QOL trajectories. Tables 3 and 4 present the full set of results for the unconditional LCGA models for youth and parent reports. Figures 1 and 2 present QOL trajectories based on estimated class means across the four time points for youth and parents, respectively. Table 5 presents the observed and estimated means and 95 % confidence intervals for the youth and parent trajectories. Analyses of youth and parent data revealed steady declines in the BIC and SSA-BIC for models with successively larger numbers of classes. This issue has been identified in previous research, showing that the BIC is very sensitive to sample size and tends to be biased toward favoring highly parameterized models [57]. As stated above, the choice of the model believed to have the optimal number of classes was based on additional considerations (e.g., LMR-LRT, entropy, posterior probabilities, parsimony) [46, 57].

**Table 3:** Optimal number of classes of quality of life (youth report)

	Number of classes (*C*)			
	1	2	3	4
Free parameters	6	9	12	15
Log likelihood	−4314.98	−4064.89	−3988.57	−3958.03
BIC	8666.42	8184.46	8050.05	8007.19
SSA-BIC	8647.38	8155.90	8011.97	7959.59
LMR-LRT	–	474.17, *p* = 0.25	144.70, *p* = 0.16	57.91, *p* = 0.30
Entropy	–	0.91	0.85	0.85
Two-class model	1	2		
1. *n*, 62 (14 %)	**0.91**	0.09		
2. *n*, 373 (86 %)	0.02	**0.98**		
Three-class model	1	2	3	
1. *n*, 315 (72 %)	**0.95**	0.05	0.00	
2. *n*, 95 (22 %)	0.13	**0.86**	0.02	
3. *n*, 25 (6 %)	0.00	0.09	**0.91**	
Four-class model	1	2	3	4
1. *n*, 17 (4 %)	**0.92**	0.06	0.02	0.00
2. *n*, 101 (23 %)	0.04	**0.83**	0.01	0.12
3. *n*, 14 (3 %)	0.05	0.02	**0.93**	0.00
4. *n*, 303 (70 %)	0.00	0.06	0.00	**0.94**

Bold values represent the posterior probability values for each class within each model

Intercepts (*I*) and slopes (*S*) for selected two-class model. C1: *I* = 18.48 (1.42), *S* = − 0.11 (0.58); C2: *I* = 26.59 (0.23), *S* = − 0.01 (0.08)

**Table 4:** Optimal number of classes of quality of life (parent report)

	Number of classes (*C*)				
	1	2	3	4	5
Free parameters	6	9	12	15	18
Log likelihood	−4394.10	−4154.06	−4071.06	−4032.76	−4014.04
BIC	8824.68	8362.84	8215.07	8156.73	8137.51
SSA-BIC	8805.64	8334.28	8176.99	8109.13	8080.39
LMR-LRT	–	455.12, *p* = 0.00	157.39, *p* = 0.03	72.60, *p* = 0.07	35.51, *p* = 0.46
Entropy	–	0.80	0.76	0.80	0.82
Two-class model	1	2			
1. *n*, 319 (73 %)	**0.95**	0.05			
2. *n*, 118 (27 %)	0.09	**0.91**			
Three-class model 1	2	3			
1. *n*, 78 (18 %)	**0.92**	0.08	0.00		
2. *n*, 206 (47 %)	0.03	**0.87**	0.10		
3. *n*, 153 (35 %)	0.00	0.12	**0.88**		
Four-class model	1	2	3	4	
1. *n*, 143 (33 %)	**0.90**	0.00	0.10	0.00	
2. *n*, 16 (4 %)	0.00	**0.89**	0.00	0.11	
3. *n*, 207 (47 %)	0.09	0.00	**0.87**	0.04	
4. *n*, 71 (16 %)	0.00	0.01	0.07	**0.92**	
Five-class model	1	2	3	4	5
1. *n*, 62 (14 %)	**0.90**	0.01	0.06	0.00	0.03
2. *n*, 14 (3 %)	0.11	**0.87**	0.00	0.00	0.02
3. *n*, 207 (48 %)	0.04	0.00	**0.87**	0.00	0.01
4. *n*, 144 (33 %)	0.00	0.00	0.10	**0.90**	0.00
5. *n*, 10 (2 %)	0.13	0.00	0.04	0.00	**0.84**

Bold values represent the posterior probability values for each class within each model

Intercepts (*I*) and slopes (*S*) for the selected three-class model. C1: *I* = 27.14 (0.14), *S* = 0.05 (0.10); C2: *I* = 22.87 (0.24), *S* = 0.05 (0.06); C3: *I* = 16.44 (0.61), *S* = 0.32 (0.30)

**Table 5:** Observed and estimated means and confidence intervals for youth and parent trajectories

Analysis	*M* (observed)	*M* (estimated)	95 % CI
Youth			
High and stable			
Time 1	26.54	26.59	26.21–26.97
Time 2	26.74	26.58	26.28–26.89
Time 3	26.46	26.57	26.30–26.85
Time 4	26.57	26.57	26.27–26.86
Moderate/low and stable			
Time 1	18.32	18.48	16.14–20.81
Time 2	18.78	18.37	16.05–20.68
Time 3	17.77	18.26	15.61–20.90
Time 4	18.31	18.14	14.91–21.38
Parent			
High and stable			
Time 1	27.08	27.14	26.91–27.38
Time 2	27.33	27.19	26.82–27.56
Time 3	27.14	27.24	26.72–27.76
Time 4	27.33	27.41	26.61–27.97
Moderate and stable			
Time 1	22.89	22.87	22.48–23.26
Time 2	22.84	22.83	22.41–23.25
Time 3	22.62	22.78	22.31–23.25
Time 4	22.87	22.74	22.19–23.28
Moderate/low and stable			
Time 1	16.38	16.44	15.44–17.43
Time 2	16.68	16.75	16.21–17.31
Time 3	17.52	17.08	16.79–17.37
Time 4	17.03	17.41	16.82–17.98

For the youth model, the overall weight of the evidence supported a two-class solution: a ‘high and stable’ QOL group (85.7 %) and a ‘moderate/low and stable’ group (14.3 %). Although the two-class solution did not have the lowest BIC value, the value did not drop substantially from the model with two classes to the model with three classes (compared to the drop in value from the single- to two-class solution). The LMR-LRT value was not significant. However, entropy was highest for the two-class solution (0.91), suggesting excellent classification quality. The two-class solution had the highest posterior probability values (0.98, 0.91). Finally, the decision to select the two-class solution over the three-class solution was based on practical considerations of statistical power. Despite having a reasonably high entropy value of 0.86, one of the classes in the three-class solution contained just 5.6 % (*n* = 25) of the total sample, ruling out a more in-depth analysis of factors associated with membership in this group. Estimated mean trajectories of the three-class solution for the youth self-report are presented in Figure A in a supplement to this paper.

For the parent model, three QOL groups were supported: ‘high and stable’ (35.7 %), ‘moderate and stable’ (46.6 %), and ‘moderate/low and stable’ (17.7 %). This decision was based on a substantially lower BIC value for the three-class solution compared to the two-class solution, an acceptable entropy value (0.76), high posterior probability values (0.92, 0.87, 0.88), and a statistically significant LMR-LRT value (157.39, *p* < 0.05). Despite having higher entropy, the four-class solution was not chosen. The drop in the BIC was not substantial, and the LMR-LRT value failed to reach statistical significance. The four-class solution also contained a class with only 15 cases, therefore limiting the ability to examine factors related to class membership.

### Multinomial logistic regression

Table 6 reports baseline factors associated with group membership for the youth report of global perceived QOL. Odds ratios are reported with the ‘high and stable’ group representing the reference group. Youth with greater emotional symptoms (OR = 1.32; *p* = 0.001) were more likely to be in the ‘moderate/low and stable’ group. Youth with reported higher spirituality (OR = 0.86; *p* < 0.001) and greater self-determination (OR = 0.88; *p* = <0.001) were less likely to be in the ‘moderate/low and stable’ group. Those youth with higher family support (OR = 0.84; *p* < 0.001) were also less likely to be in the ‘moderate/low and stable’ group. In addition, youth with higher school productivity/engagement (OR = 0.80; *p* < 0.05) and greater school belongingness/safety (OR = 0.88; *p* < 0.05) were less likely to be in the ‘moderate/low and stable’ group compared to the ‘high and stable’ group.

**Table 6:** Logistic regression of correlates of group membership for youth QOL (youth report)

Correlates	C1 (vs C2)				
	Est (SE)	OR	95 % CI	*p*	ES
Youth functioning/personal factors					
Emotional symptoms (YR)	−0.28 (0.08)	1.32	1.06–1.64	0.001	0.15
Pain/other physical symptoms (PR)	−0.32 (0.20)	1.38	0.82–2.34	0.11	0.18
Self-determination (YR)	0.12 (0.04)	0.88	0.81–0.96	<0.001	−0.07
Spirituality (YR)	0.15 (0.04)	0.86	0.77–0.95	<0.001	−0.08
School productivity/engagement (PR)	0.21 (0.08)	0.80	0.61–1.06	0.04	−0.12
Environmental factors					
Family social support for youth (YR)	0.16 (0.04)	0.84	0.78–0.93	<0.001	−0.10
Overall family functioning (PR)	0.01 (0.03)	0.99	0.91–1.07	0.51	0.01
School belongingness/safety (YR)	0.12 (0.06)	0.88	0.76–1.03	0.03	−0.07
Home and community barriers (PR)	−0.03 (0.03)	1.03	0.97–1.10	0.21	0.02

Results adjusted for youth and parent age, youth and parent gender, youth age at diagnosis, parent marital status, education, and income. Results adjusted for design effects (youth nested within centers)

*YR* youth report, *PR* parent report

Entropy for conditional model = 0.92

C1 = moderate/low and stable quality of life (15.7 % *n* = 68)

C2 = high and stable quality of life (reference group) (84.3 % *n* = 367)

*n* = 435 youth; 8 youth treatment centers

Table 7 reports baseline factors associated with group membership for the parent report. Odds ratios are reported with the ‘high and stable’ group once again representing the reference group. Youth with higher emotional symptoms (OR = 1.20; *p* = 0.001; OR = 1.55; *p* < 0.001, respectively) and more pain/other physical symptoms (OR = 1.38; *p* < 0.01; OR = 1.54; *p* < 0.01, respectively) were more likely to be in the ‘moderate and stable’ group or the ‘moderate/low and stable’ group compared to the ‘high and stable’ group. Youth with higher spirituality (OR = 0.88; *p* < 0.05) were less likely to be in the ‘moderate/low and stable’ group. Youth with higher school productivity/engagement were also less likely to be in the ‘moderate and stable’ or ‘moderate/low and stable’ groups (OR = 0.69; *p* < 0.001; OR = 0.67; *p* < 0.001, respectively). Youth with reported higher family functioning (OR = 0.87; *p* < 0.001) were less likely to be in the ‘moderate/low and stable’ group. Finally, those with more home/community barriers (OR = 1.16; *p* < 0.001; OR = 1.34; *p* = 0.001, respectively) were more likely to be in the ‘moderate and stable’ group or the ‘moderate/low and stable’ group compared to the ‘high and stable’ group.

**Table 7:** Logistic regression of correlates of group membership for youth QOL (parent report)

Correlates	C2 (vs C3)					C1 (vs C3)				
	Est (SE)	OR	95 % CI	*p*	ES	Est (SE)	OR	95 % CI	*p*	ES
Youth functioning/personal factors										
Emotional symptoms (YR)	0.19 (0.05)	1.20	1.05–1.37	0.001	0.10	0.44 (0.06)	1.55	1.33–1.81	<0.001	0.24
Pain/other physical symptoms (PR)	0.32 (0.12)	1.38	1.01–1.89	0.008	0.18	0.43 (0.16)	1.54	1.02–2.32	0.007	0.24
Self-determination (YR)	–0.01 (0.06)	0.99	0.85–1.17	0.98	–0.01	–0.05 (0.07)	0.94	0.81–1.09	0.29	−0.03
Spirituality (YR)	–0.04 (0.02)	0.96	0.90–1.02	0.07	–0.02	–0.06 (0.06)	0.88	0.83–1.02	0.04	−0.07
School productivity/engagement (PR)	–0.36 (0.07)	0.69	0.58–0.83	<0.001	–0.21	–0.40 (0.10)	0.67	0.52–0.88	<0.001	−0.22
Environmental factors										
Family social support for youth (YR)	–0.03 (0.06)	0.97	0.84–1.12	0.62	–0.02	–0.12 (0.09)	0.89	0.69–1.13	0.20	−0.07
Family functioning (PR)	–0.04 (0.04)	0.96	0.87–1.07	0.38	–0.02	–0.13 (0.03)	0.87	0.81–0.94	<0.001	−0.08
School belongingness/safety (YR)	0.15 (0.10)	1.16	0.90–1.50	0.13	0.08	0.14 (0.07)	1.15	0.94–1.40	0.06	0.08
Home and community barriers (PR)	0.15 (0.03)	1.16	1.06–1.28	<0.001	0.08	0.28 (0.09)	1.34	1.06–1.67	0.001	0.16

Results adjusted for youth and parent age, youth and parent gender, youth age at diagnosis, parent marital status, education, and income. Results adjusted for design effects (youth nested within centers)

*YR* youth report, *PR* parent report

Entropy for conditional model = 0.80

C1 = moderate/low and stable quality of life (15.7 % *n*, 69)

C2 = moderate and stable quality of life (48.1 % *n*, 210)

C3 = high and stable quality of life (reference group) (36.2 % *n*, 158)

*n* = 437 parents; 8 youth treatment centers

The logistic regression results in Tables 6 and 7 provide additional confirmation of the choice of the optimal number of classes for the youth and parent models. The vast majority of the hypothesized predictors of group membership were in the expected direction, evidence of the external validity of the extracted latent classes. The results increase confidence that the classes extracted from the unconditional models were not an artifact of the data, but represented qualitatively different underlying subpopulations of youth [58].

## Discussion

### QOL trajectories

This study identified unique group QOL trajectories within both the youth and parent analyses. All trajectories appeared stable over 3 years of follow-up. This is notable since study participants were passing through early to late adolescence where they would be thought to be experiencing multiple transitions, such as transferring to another school level, desiring greater independence from family, connecting more with peers, and looking to venture out into the community [12, 59]. It might be anticipated this would lead to formation of groups with changing trajectories of QOL, either increasing for those who are successfully dealing with transitions, or shifting negatively for those who are not. In addition, a sudden change in health status might be expected to result in changing trajectories. Indeed, research with individuals who had a recent onset and diagnosis of a disease or a life-threatening illness has found these types of patterns [25, 32, 60]. In contrast, the stability and lack of change in the trajectories identified here may be related to the long-term nature of youths’ conditions. In this study, the vast majority of youth had been diagnosed with a non-progressive disease before 4 years of age (77 %), with a mean age of diagnosis at 3.6 years (*SD* = 1.7). Ahmed et al. [61] showed that among individuals experiencing small changes in health status, shifts in response to a HRQOL measure were nonsignificant over a year.

Yet, distinct groups exist within the study sample, with varying levels of QOL. Youth perspectives revealed a large ‘high and stable’ and a small ‘moderate/low and stable’ group. Parent perspectives revealed medium-sized ‘high and stable’ and ‘moderate and stable’ groups, and a small ‘moderate/low and stable’ group. Two distinct trajectories in the youth analysis and three in the parent analysis can be understood within the context of existing literature. In most studies of HRQOL or perceived global QOL (including this study), parents tend to score their youths’ QOL lower than do youth [37, 62]. In most studies, the majority of parents report moderate levels of QOL for their youth. While almost a third of the parents in this study viewed their youths’ perceived QOL as high, the majority took a more moderate view. This tendency for parents to be cautious about high ratings could be related to their interpretation of specific environmental factors as being detrimental to their youths’ QOL, as found in this study. Also, they may feel concern for their youth with respect to their condition and assume that this has substantial impact on their QOL. Obtaining parent information provides a valuable supplemental view of life quality for youth and the factors that contribute to it.

Researchers suggest that lower life satisfaction among some youth with chronic conditions may not be an intrinsic function of their disease, but rather that exposure to adversity and lack of resources may account for this poorer perspective on life [28, 63]. Cummins put forth and demonstrated a theory of homeostasis that posits humans have a ‘set-point’ with respect to subjective well-being, with set-points normally ranging from 70 to 90, with a mean score of 80 on a standard 0–100 point range [64, 65]. If a group population mean drops to between 51 and 69, it may represent homeostatic failure. That is, when excessive demands are placed on an individual or groups of individuals, homeostasis can be overwhelmed. Moreover, when demands are ongoing, the homeostatic drop can become a stable and ongoing phenomenon. Group means at or below 50 are considered to represent a high risk for psychopathology [66].

For individuals or groups of individuals operating below their set-point, interventions aimed at improving aspects of life that are lowering overall life satisfaction may serve to restore homeostasis. Tomyn et al. [67] tested a number of predictions based on homeostasis theory about intervention outcomes for a sample of 4243 youth with various physical and psychosocial problems. Youths’ subjective well-being was measured using the Personal Well-Being Index—School Children [68] converted to a metric ranging from 0 to 100 points. The researchers hypothesized that youth would have varying levels of subjective well-being, and that those functioning within a normal set-point range would at best achieve a small increase from an intervention. Alternatively, for those experiencing homeostatic failure, it was hypothesized that an intervention would raise their subjective well-being substantially, potentially to the point of reaching homeostatic control. Their study confirmed these hypotheses. Although concerted universal efforts directed at building personal resources and strengthening relationships for all young people are important, Tomyn et al. [67] results highlight the need for targeted programs for those experiencing the greatest threats to their QOL to try to shift them to a higher trajectory, as it appears these groups of individuals benefit most from additional resources and supports.

### Factors related to group trajectories

Most youth in this study reported ‘high and stable’ global perceived QOL. Both the youth and parent reports of youths’ QOL point to differences in supports and resource availability for the identified ‘moderate and stable’ and ‘moderate/low and stable’ groups compared to the ‘high and stable’ groups. Multiple contextual factors were related to group membership, indicating the benefits of a comprehensive approach to assessment and intervention to enhance QOL for youth with chronic conditions. In addition, the types of factors associated with ‘moderate and stable’ and ‘moderate/low and stable’ group membership are amenable to change.

Emotional symptoms were associated with membership in other groups compared to the ‘high and stable’ group for both the youth and parent analyses. Population-based studies indicate children and youth with chronic conditions are twice to three times as likely to be reported as having mental health issues compared to those with no conditions [69, 70]. Mental health problems can often be overlooked in youth with chronic conditions and are often related to the development of psychosocial issues such as isolation and bullying [71, 72]. The parent analysis indicated pain/other physical symptoms were associated with ‘moderate and stable’ and ‘moderate/low and stable’ group membership. Research indicates long-lasting pain among children and youth with chronic conditions is often not identified or sufficiently treated [73, 74]. This study and other research suggest pain/other physical symptoms and emotional problems can have long-standing effects on QOL for a significant number of individuals and should always be inquired about in initial assessments and carefully monitored and treated throughout childhood and adolescence.

Youth with higher spirituality, described in the study questionnaire as ‘any deep feelings or beliefs’ a youth may have, were less likely to be in the ‘moderate/low and stable’ groups for both the youth and parent analyses. Listening to youth and communicating effectively to understand the importance of personal meaning in their lives may prove helpful in the process of identifying those youth who are experiencing ongoing poor QOL [75, 76]. The value of developing clinical listening and communication skills among service providers for appreciating clients’ situations and understanding their worldview is supported within the literature [77].

Moreover, this research supports the position that provision of family-centered services that include an emphasis on family well-being is integral to the life quality of youth with chronic conditions as they go through adolescence [78]. Youth with higher family social support were less likely to be in the ‘moderate/low and stable’ group in the youth analysis, while youth with higher family functioning were less likely to be in the ‘moderate/low and stable group’ in the parent analysis. More barriers at home such as family stress and more community barriers such as lack of services and funding were associated with membership in the ‘moderate and stable’ and the ‘moderate/low and stable’ groups in the parent analysis. Routine assessment of family well-being and community barriers in childhood and adolescence by service providers could help to identify those children and their families who require additional supports and resources.

Youth with higher school productivity and engagement were less likely to be in the ‘moderate and stable’ group in the parent analysis or in the ‘moderate/low and stable’ groups in the youth and parent analyses. Similarly, youth with a greater sense of school belongingness and safety and higher self-determination in terms of goal orientation were less likely to be in the ‘moderate/low and stable’ group in the youth analysis. Other research indicates an important link between self-determination and QOL for youth with chronic conditions [12, 79, 80]. Universal prevention programs may be important for promoting inclusive school cultures and for maintaining high QOL for youth who are doing well at school, while those who are experiencing difficulties with learning, motivation, and being accepted at school may benefit most from targeted best practice interventions that are available from early childhood.

### Study strengths, limitations and future research

To the authors’ knowledge, this is the first paper to identify distinct trajectories of both youth and parent reports of youths’ global perceived QOL over a period of time for youth with chronic health conditions and to examine factors associated with those trajectories. The factors identified may represent opportunities for intervention to enhance unfavorable QOL trajectories. The study is strengthened given its alignment with homeostasis theory [65, 67]. The relatively large sample size increased study validity. Moreover, the youth and parent measures of global perceived QOL, as used in this study, were shown to have good psychometric properties [37].

This study has several limitations. Results are specific to eight children’s treatment centers in Ontario, Canada. Thus, trajectories identified in this study will need to be cross-validated using independent samples. Future research is needed that uses multilevel modeling where center-level variables are considered in the trajectory analysis. The use of 12-month follow-up intervals may have limited the sensitivity of the change evaluation. Upcoming studies might use shorter intervals to capture upward and downward trends that may exist in QOL due to episodic issues of a health or social nature. As well, studies that follow youth over longer time periods are needed, allowing examination of QOL trajectories as they move into adulthood.

Two of the nine measures of study correlates had low internal consistency estimates. Therefore, caution should be exercised when interpreting results related to the constructs those measures represent (i.e., self-determination, pain/physical symptoms). Self-reported and parent-reported measures were used for hypothesized correlates and as well as global perceived QOL in the youth and parent analyses, potentially leading to bias and shared method variance problems. While the primary purpose of this research was to examine youth QOL and the factors that influence it from the perspectives of youth and their parents, additional reports of youths’ mental and physical well-being, school productivity, the home, school, and community environment from other sources may have served to strengthen the study.

The initial response rate was low (31 %). However, as described, participating families did not differ significantly on a number of socio-demographic characteristics from those who declined to participate, with the exception of parent age. For 31 % of families, the youth, parent, or both missed completing at least one follow-up interview, potentially affecting the validity of results. Still, as stated, no significant correlates of youth or parent non-completion of at least one or all follow-up interviews emerged from logistic regression analyses, lessening bias concerns.

Previous research has indicated the primary importance of paternal support to changes in QOL for youth with congenital heart disease [27]. Upcoming studies should parse out the differential effects of maternal, paternal, and sibling support on QOL trajectories. In this study, factors were entered into the LCGA models as additive main effects. Future research should focus on examining mediators and moderators that might contribute to trajectories.

## Electronic supplementary material

Below is the link to the electronic supplementary material.

Supplementary material 1 (DOCX 116 kb)
