# Supplementary material for: Three-year trajectories of global perceived quality of life for youth with chronic health conditions
Source: Qual Life Res. 2016 Jul 5;25(12):3157–71. doi: 10.1007/s11136-016-1353-z (PMC5102979; doi:10.1007/s11136-016-1353-z)
Supplement: Supplementary file 1 — Supplementary material 1 (DOCX 116 kb) [file 11136_2016_1353_MOESM1_ESM.docx]

**Supplemental Fig. A** Unconditional Model of Distinct Trajectories for Three-Class Model - Youth Report
